# Splice-altering variant of *PJVK* gene in a Mauritanian family with non-syndromic hearing impairment

**DOI:** 10.1007/s13353-024-00903-x

**Published:** 2024-09-04

**Authors:** Malak Salame, Crystel Bonnet, Amrit Singh-Estivalet, Selma Mohamed Brahim, Solene Roux, Ely Cheikh Boussaty, Mouna Hadrami, Cheikh Tijani Hamed, Abdellahi M’hamed Sidi, Fatimetou Veten, Christine Petit, Ahmed Houmeida

**Affiliations:** 1 Unité de Recherche Sur Les Biomarqueurs Dans La Population Mauritanienne, UN-FST, Nouakchott, Mauritania; 2 Institut de L’Audition, Institut Pasteur, Inserm, Paris, France; 3 Centre National d’Oncologie (CNO), Unité de Recherche Et d’Enseignement, Nouakchott, Mauritania; 4 Institut d’Hépato-Virologie, Nouakchott, Mauritania; 5 Collège de France, Paris, France; 6 Division of Otolaryngology, Department of Surgery, University of California, 9500 Gilman Drive, Mail Code 0666, La Jolla, San Diego, CA 92093 USA

**Keywords:** *PJVK*, Hearing impairment, Splice-site acceptor, Minigene, Mauritania

## Abstract

*PJVK* gene was recently shown to create hypervulnerability to sound in humans and was the first human gene implicated in non-syndromic hearing impairment due to neural defect. Targeted next-generation sequencing of over 150 known deafness genes was performed in the proband. Sanger sequencing was used to validate the *PJVK* variant and confirm familial segregation of the disease. A minigene-based assay has been performed to assess the impact of the variant on splicing. We identified a novel c.550-6A > G acceptor splice-site variant in the *PJVK* gene in the homozygous state in a Mauritanian child with severe to profound congenital deafness. The substitution was located in intron 4. The effect of the variation was demonstrated by a minigene assay which showed that the variation, an insertion of an additional 5 bp, created a new splice site resulting in the appearance of a premature stop codon (p.Phe184Tyrfs*26) and likely a truncated protein. This result constitutes a new splice-site variant report in the *PJVK* gene leading to DFNB59 type associated with autosomal recessive non-syndromic hearing impairment (ARNSHI).

## Introduction

Non-syndromic hearing impairment (NSHI) are essentially sensorineural, as they are often associated with lesions of the inner ear structures. Their mode of inheritance is 80% autosomal recessive (DFNB) but can be also autosomal dominant (DFNA), X-linked (DFNX), or mitochondrial (Smith et al. 2005; Morton 2014). More than 150 genes such as *GJB2*, *SLC26A4*, *TECTA*, *PJVK*, *COL11A2*, *MYO15A*, *TMC1*, and *RDX* have been associated with genetic heterogeneity (Camp and Smith 2024). The *PJVK* gene (MIM #610219) is located at locus 2q31.2 and contains 7 exons spanning a genomic region of 9950 bp. Variants of this gene have been associated with multiple cases of autosomal recessive (AR) NSHI (Domínguez-Ruiz et al. 2022). Alterations in the *PJVK* gene have also recently been shown to result in hypervulnerability to sound in humans and mice, probably due to elevated levels of oxidative stress in inner ear hair cells (Defourny et al. 2019). In addition, the pejvakin protein encoded by the *PJVK* gene has been implicated in neuronal signal transmission and has been shown to be necessary for the proper functioning of neurons in the auditory pathway (Delmaghani et al. 2006; Zhang et al. 2015a). Unlike the previously described sensorineural deafness genes, all of which underlie cochlear cell pathologies, DFNB59 was the first human gene implicated in non-syndromic deafness due to neural defect (Delmaghani et al. 2006).

Testing for the *GJB2* gene is commonly carried out as a primary screening test for any hearing impairment. Variations in this gene are indeed the most common cause of hereditary congenital hearing impairment in many countries, and are present in around half of people with severe to profound congenital ARNSHI. We have previously reported four pathogenic variants: (c.35delG and c.94C > T) in the *GJB2* gene, c.179T > C in the *LRTOMT* gene and (c.1015C > T) in the *CDC14A* gene in Mauritanian children with non-syndromic hearing impairment (Moctar et al. 2016; Salame et al. 2023; Delmaghani et al. 2016). Given these concordant data on the involvement of *PJVK* gene in hearing impairment, we next investigated the implication of this gene in Mauritanian patients negative for the *GJB2* and *LRTOMT* genes. We report here the identification of a novel splice site pathogenic variant in the *PJVK* (DFNB59) gene in a Mauritanian child affected by prelingual non-syndromic hearing impairment.

## Materials and methods

### Targeted exome sequencing

Genomic DNA extraction, from blood samples collected in EDTA tubes, was performed for 30 families with prelingual deafness (one patient selected per family), using the Qiagen DNA Blood Minikit procedure (QIAGEN Genomic DNA Handbook.2015). We followed a high-throughput targeted exome sequencing strategy, called Hear panel, to simultaneously analyze all coding exons and bordering intronic sequences of 163 genes known to be involved in non-syndromic or syndromic hearing impairment (Table 1). Library preparation, sequence capture, sequencing, and data analysis were performed by IntegraGen SA (Evry, France) on HiSeq 2000 sequencer (Illumina). Bioinformatics analysis of sequencing data was based on the Illumina pipeline (CASAVA1.8.2).

**Table 1:** List of genes targeted in heat panel

ABHD12	CLDN14	DIAPH1	GJB2	KCNQ1	MYO3A	PNPT1	SMPX
ACTG1	CLDN9	DIAPH3	GJB3	KCNQ4	MYO6	POU3F4	SOX10
ADCY1	CLIC5	DMXL2	GJB4	KITLG	MYO7A	POU4F3	SPNS2
ADGRV1	CLPP	DSPP	GJB6	LARS2	NARS2	PPIP5K2	STRC
AIFM1	CLRN1	EDN3	GPSM2	LHFPL5	NLRP3	PRPS1	SYNE4
ARSG	CLRN2	EDNRB	GRAP	LMX1A	OSBPL2	PTPRQ	TBC1D24
ATP6V1B1	COCH	ELMOD3	GRHL2	LOXHD1	OTOA	RDX	TECTA
BDP1	COL11A1	EPS8	GRXCR1	LRTOMT	OTOF	REST	TJP2
BSND	COL11A2	EPS8L2	GRXCR2	MAP1B	OTOG	ROR1	TMC1
C10ORF2	COL2A1	ERAL1	GSDME	MARVELD2	OTOGL	S1PR2	TMEM132E
CABP2	COL4A3	ESPN	HARS2	MCM2	P2RX2	SCD5	TMIE
CCDC50	COL4A4	ESRP1	HGF	MET	PAX3	SERPINB6	TMPRSS3
CD164	COL4A5	ESRRB	HOMER2	METTL13	PCDH15	SIX1	TNC
CDC14A	COL4A6	EYA1	HSD17B4	MIRN96	PDE1C	SIX5	TPRN
CDH23	COL9A1	EYA4	IFNLR1	MITF	PDZD7	SLC12A2	TRIOBP
CEACAM16	COL9A2	FAM65B/RIPOR2	IKZF2	MPZL2	PEX1	SLC17A8	TRRAP
CEP78	COL9A3	FDXR	ILDR1	MSRB3	PEX6	SLC22A4	TSPEAR
CEP250	CRYM	FOXI1	KARS	MYH14	PHYH	SLC26A4	TUBB4B
CHD7	DCDC2	GAB1	KCNE1	MYH9	PJVK	SLC26A5	USH1C
CIB2	DIABLO	GIPC3	KCNJ10	MYO15A	PLS1	SLITRK6	USH1G
WBP2	WFS1	WHRN					

CASAVA cascade included alignment of sequencing data to the reference human sequence (hg19 reference genome), allele calling, and association with their position, depth, base count, associated genotypes, homozygous/heterozygous status, and associated quality scores.

### Sanger sequencing and segregation analysis

Sanger sequencing was used to validate the *PJVK* variant (NM_001042702.5) in the child and confirm its familial segregation. Primers were designed with Primer3 (https://primer3.ut.ee/) as follows: forward 5′-CAGGAGTTTCTGTTGGACCA-3′ and reverse 5′-TGCAGACCCTTAACTCACCA-3′.

PCR reactions (20 µl) contained 1 µl (20–30 ng) of genomic DNA, 1 µl (10 µM) of each specific primer, 10 µl of AmpliTaq Gold 360 Master Mix (Life Technologies) and 7 µl of distilled water. The PCR program featured a DNA denaturation step at 95 °C for 10 min followed by 35 amplification cycles (denaturation at 94 °C for 30 s, annealing at 53 °C for 35 s, and extension at 72 °C for 40 s) tailed by a 5-min final extension at 72 °C. PCR products were sequenced on capillary ABI3730 Genetic Analyzer (Applied Biosystems, CA, USA). Data obtained were then matched with reference sequences of the *PJVK* gene using Seqscape 3.0 software program package (Gene Codes, MI, USA).

### Minigene splicing assay

The minigene splicing assay used here to evaluate the impact of the variant detected at the splice site of intron 4 of the *PJVK* gene was done as previously described (Cooper 2005). This assay was performed on the patient carrying the variation and a health control (wild type (WT)). The targeted genomic sequence (between exons 4 and 5 of DFNB59 and spanning 4438 bp) was amplified by PCR using Phusion DNA polymerase (High-Fidelity DNA Polymerase) with gene-specific primers containing restriction enzyme sites (XhoI and BamHI): forward 5′-TTCCTCGAGCCAGATCAAGAATGGCAGCC-3′ and reverse 5′-GGATCCTTCCTGATCCCTTGGGCTAC-3′. Subsequently, the PCR fragment was ligated into the pET01b vector. The ligation product (recombinant plasmid) was transformed into TOP10 or Stellar (*Escherichia coli*) competent cells and positive clones were selected then sequenced by the Sanger method to verify and confirm clone genotypes. After sequence alignment, the best plasmids (wild-type and mutants) were selected for transfection into eukaryotic cell lines.

#### Transfection in eukaryotes COS-7 cells

Wild-type and mutant minigenes were transfected in triplicate into COS-7 cells using Lipofectamine™ 2000 transfection reagent (Invitrogen). COS-7 are fibroblast-like cell lines derived from monkey kidney tissue. They were used here because of their high transfection efficiency.

Cells were harvested 48 h after transfection and total RNA was extracted using the QIAGEN mini kit.

#### Reverse transcription PCR

The cDNA was obtained from 500 ng of extracted RNA by following the RT-PCR protocol with Superscript™ III Reverse Transcriptase (Invitrogen Ref: 18,080–085) using oligo dT. PCR amplifications were generated by Phusion DNA polymerase using primers specific to the native 5′ and 3′ exons of the pET01b vector: ETPR04 5′-GGATTCTTCTACACACCC-3′ and ETPR05 5′-TCCACCCAGCTCCAGTTG-3′. The amplification was performed for 30 cycles of 95 °C for 30 s, 55 °C for 45 s, and 72 °C for 1 min. The PCR products were then visualized on a 1% agarose gel and the band of interest was cut out. Gel PCR products were extracted and sequenced by Sanger.

## Results

### Variant identification

**Fig. 1:**
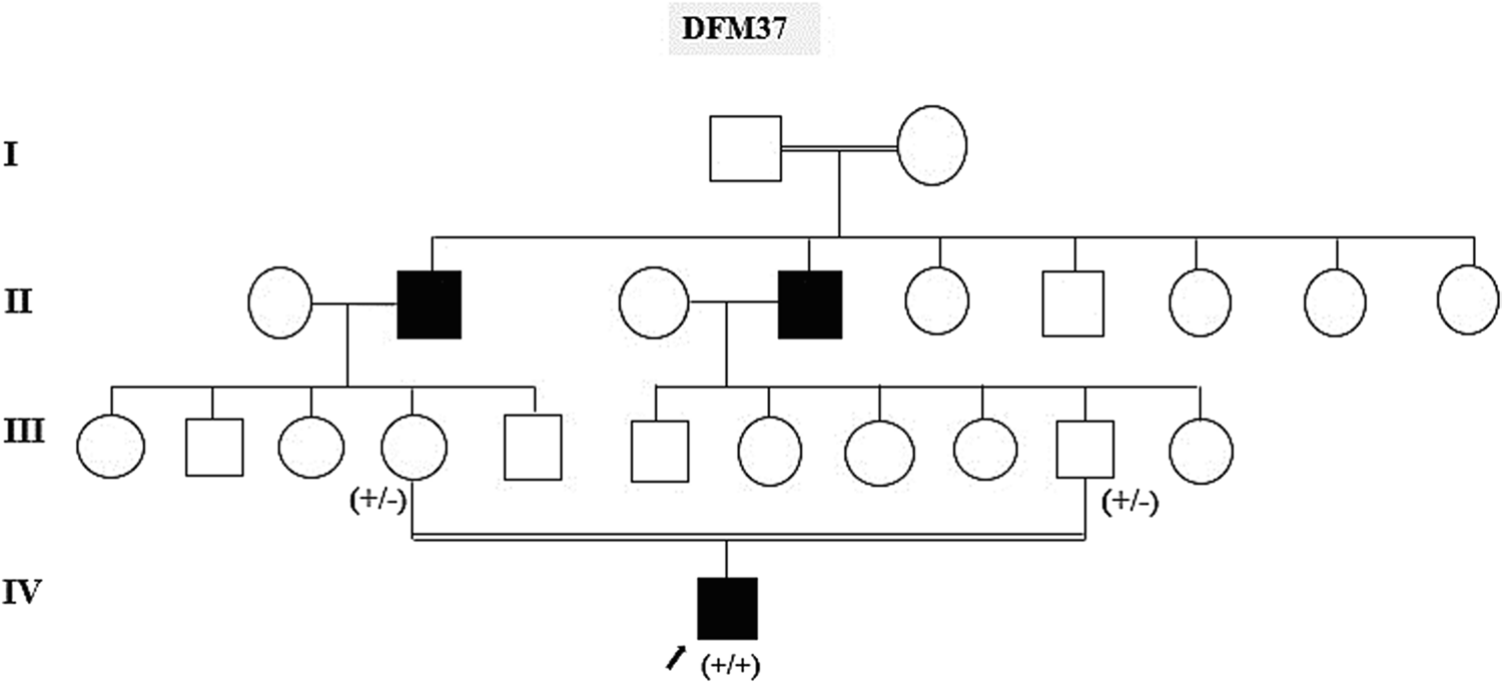
Pedigree of the Mauritanian family of patient DFM37 with non-syndromic deafness (autosomal recessive pattern). Clear symbols, healthy individuals; filled symbols, deaf individuals. The arrow indicates DFM37 patient (IV 1)

**Fig. 2:**
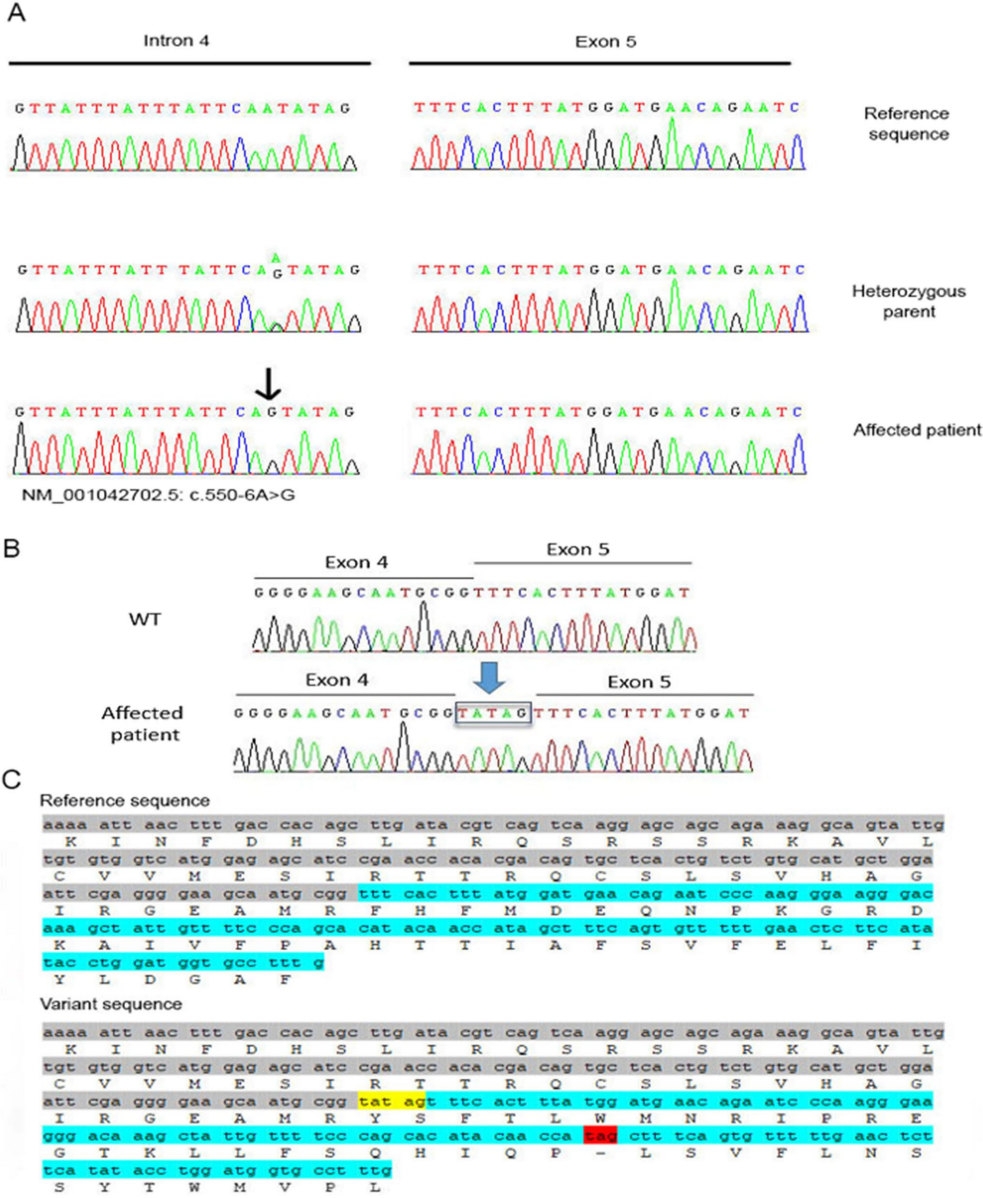
Genetic analysis of c.550-6A > G variant in the *PJVK* gene: **A** Confirmation by Sanger sequencing of electropherograms showing the reference sequence and the variant in heterozygous and homozygous states in intron 4. The arrow indicated the position of the mutated nucleotide. **B** Sequencing results of minigene constructs revealed the addition of 5 bp and the creation of a new splicing site. **C** Nucleotide and protein sequences for wild-type and affected patient. The addition of 5 pb (yellow) in the patient sequence created a new splice site inducing the appearance of the premature stop codon (red)

Targeted high-throughput exome sequencing (hear panel: Table 1) on patient DNA samples (ARNSHI) in which no variants were found in *GJB2* and *LRTOMT*, showed, after the filtering procedure, a new variant of the *PJVK* gene NM_001042702.5 (*PJVK*): c.550-6A > G (Chr2(GRCh37): g.179323231A > G) identified in a 16-year-old boy suffering from prelingual deafness. The DNA change, a substitution at the splice site in intron 4 (exon 5 acceptor site) is present in the homozygous state in the proband (Fig. 1). The variant was predicted as pathogenic using Alamut 2.15.0 visual prediction software which compiles data from splicing defect prediction algorithms (MaxEnt, Gene splicer, Human Splicing Finder, NNSPLICE). The scores of both sites, the canonical site, and the novel splice site, respectively, are shown in Table 2. Sanger sequencing also performed in healthy parents confirmed the c.550-6A > G variant in the child and showed its segregation in the heterozygous state performed in the healthy parents (Fig. 2A). No known pathogenic biallelic variants were detected in the other panel genes.

**Table 2:** Splicing prediction scores in the reference (canonical) and new splicing sites

	Splice Site Finder like [0–100]	MaxEntScan [0–16]	NNSplice [0–1]	Gene Splicer [0–21]
Reference sequence	78.78	4.91	0.81	-
c.550-6A > G	86.81	5.77	-	2.54

### Minigene assay

Visualization of wild-type and mutant minigene constructs showed that the A-to-G transition at the exon 5 acceptor site resulted in a novel splice site with an additional 5 bp present in mutants (337 bp band). Sanger sequencing confirmed the genotypes of the identified clones, all minigene constructs were found mutated and therefore no normal splicing was detected. Sequence alignment showed that the c.550-6A > G variation resulted in alternative splicing leading to the appearance of a premature stop codon and hence a truncated protein (c.550-6A > G, p. Phe184Tyrfs*26) (Fig. 2B).

### Clinical features of the patient

Among the selected patients (ARNSHI) for whom no variants were found in *GJB2* and *LRTOMT*, a 16-year-old boy with prelingual deafness was carefully studied as he was the only family member carrying the variant in the homozygous state. The parents were consanguineous, and both had normal hearing. Other cases of deafness were reported in the family (II1 and II2) (Fig. 1). The segregation of the phenotype was compatible with autosomal recessive inheritance indicating a form of DFNB deafness (Fig. 1). Severity of the hearing impairment, measured by pure tone audiometry, at frequencies of 0.25, 0.5, 1, 2, and 4 kHz in ambient air conditions for both right and left ears, was assessed using the following hearing thresholds in decibels hearing impairment: HI normal (< 20 dB), mild (20 < 40 dB), moderate (40 < 70 dB), severe (70 < 90 dB), and profound (≥ 90d B).

**Fig. 3:**
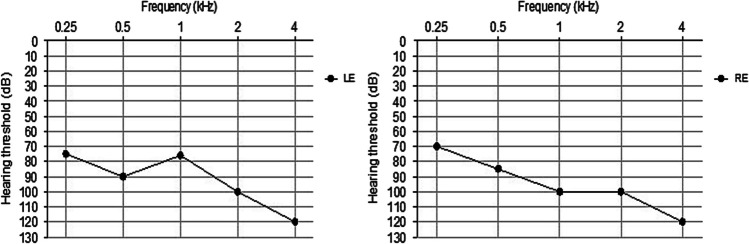
Pure tone audiometry for patient DFM37 at the age of 16 years old. RE, right ear; LE, left ear. The vertical axis indicates the sound level in decibels (dB) and the horizontal axis shows frequency in Hertz (Hz). Audiograms of both ears represent air conduction

The characteristic of hearing phenotype is shown in Fig. 3. Air conditions data at 0.5, 1, and 2 kHz were used to calculate the average hearing threshold (96.6 dB HL) in both right and left ears (Fig. 3) and revealed a profound bilateral sensorineural hearing impairment.

## Discussion

Targeted exome sequencing (hear panel) has become one of the most effective and widely developed molecular methods to investigate genetic variants associated with hearing impairment. Using this technology, we detected a novel variant (c.550-6A > G) located in intron 4 of the *PJVK* gene in a Mauritanian family. Although this variant has already been cited in the NCBI database (rs1158151073), our result is the first report highlighting its involvement in hearing impairment. It was confirmed by Sanger sequencing and found in the homozygous state in the proband patient (IV:1). The change was segregated with profound autosomal recessive sensorineural hearing impairment as shown by clinical examination and audiogram graph.

Although over 150 genes have been associated with NSHI, the explicit correlation between genotype and disease remains to be determined, as few of affected patients carry these genetic variants (Camp and Smith 2024). To date, 23 alterations in the *PJVK* gene including truncating variants, missense variants, and in-frame deletions have been associated with ARNSHI in families of diverse geographical origins (Domínguez-Ruiz et al. 2022; Ebermann et al. 2007a; Richard et al. 2019a; Bademci et al. 2016a). In this study, we reported the first *PJVK* variant associated with autosomal recessive non-syndromic in the Mauritanian population. The variant was found only in one family (3.4%) out of the 29 GJB2- and LRTOMT-negative families examined. This finding is in line with the overall low prevalence of variants in this gene in ARNSHI patients (Collin et al. 2007a). For instance, variations in DFNB59 have been associated with autosomal recessive non-syndromic hearing impairment in about 6.7% of *GJB2*-negative Pakistani families. Pathogenic *PJVK* variants were also rare in Caucasian populations with only 1 case out of 140 families (0.7%) with ARNSHI (Camp and Smith 2024; Castillo et al. 2022).

The protein encoded by the *PJVK* gene, a member of the gasdermin family, contains a nuclear localization signal (residues 249–258) and a zinc-binding motif (residues 305–331) in the C-terminal domain. It has been found to play an important role in the activity of auditory pathway neurons (Collin et al. 2007b; Roux et al. 2006). Previous studies have also shown that *PJVK* variants affected cell signaling in hair cells and sensory neurons and appeared to cause severe to profound hearing impairment (Ebermann et al. 2007b).

Including the novel variant, 24 pathogenic variants of this gene have so far been reported (Table 3). As shown by our patient, most of these variants were in the homozygous state in patients and were largely (14/24) protein-truncating variants (Table 3).

**Table 3:** Pathogenic variants reported to date in *PJVK* gene (NM_001042702.5) and associated phenotypes. Report from Deafness Variation Database (https://deafnessvariationdatabase.org/gene/PJVK)

Exon	Variants	Protein Level	Feature of the Hearing Loss	Population	Reference
2	c.113dep	p.Lys41Glufs*8	Severe to profound	Morocco	Ebermann et al. 2007a)
2	c.122del	p.Lys41Serfs*8	Profound	Iran	Schwander et al. 2007)
2	c.147T > A	p.Tyr49*	NR	Pakistan	Khan et al. 2019)
2	c.158C > G	p.Ser53*	NR	Pakistan	Richard et al. 2019a)
2	c.161C > T	p.Thr54Ile	Severe	Iran	Delmaghani et al. 2006)
2	c.162_172del	p.Pro55fs*23	NR	Pakistan	Richard et al. 2019b)
Intron 2	c.211 + 1G > T	p ?	NR	Iran	Sloan-Heggen et al. 2015)
3	c.274C > T	p.Arg92*	Stable/severe to profound	Iran	Sloan-Heggen et al. 2015)
3	c.406C > T	p.Arg136*	Stable/moderate to severe	Morocco/Pakistan	Salime et al. 2017; Zhou et al. 2020)
4	c.485G > A	p.Ser162Asn	Profound	Pakistan	Bademci et al. 2016b)
4	c.499C > T	p.Arg167*	Severe to profound	Iran/Turkey	Collin et al. 2007c; Yan et al. 2016)
4	c.547C > T	p.Arg183Trp	Severe to profound	Iran/Turkey	Chaleshtori et al. 2007; Zhang et al. 2015b)
Intron 4	c.550-6A > G	p.Phe184Tyrfs*26	Profound	Mauritania	This study
6	c.671T > G	p.Leu224Arg	Stable, profound	Spain	Domínguez-Ruiz et al. 2022)
6	c.726del	p.Phe242Leufs*7	NR	Iran	Chaleshtori et al. 2007)
6	Deletion of whole exon		NR	Iran	Sloan-Heggen et al. 2015)
7	c.880del	p.His294Ilefs*43	Stable, profound	Spain	Domínguez-Ruiz et al. 2022)
7	c.880C > G	p.His294Asp	Stable, profound	Italy	Domínguez-Ruiz et al. 2022)
7	c.908_910del	p.Asn303del	NR	Pakistan	Richard et al. 2019b)
7	c.930_931del	p.Cys312Trpfs*19	Severe to profound	China	Zhang et al. 2015b)
7	c.950del	p.Phe317Serfs*20	Profound	Italy	Domínguez-Ruiz et al. 2022)
7	c.970G > T	p.Gly324Trp	Severe to profound	Iran	Sloan-Heggen et al. 2015)
7	c.988del	p.Val330Leufs*7	Profound	Iran	Chaleshtori et al. 2007)
7	c.1028G > C	p.Cys343Ser	Stable, profound	Pakistan	Mujtaba et al. 2012)

The exons of a gene are joined in different combinations, leading to different but related mRNA transcripts, a cellular process known as alternative splicing. The regulation of cellular DNA transcription can be disrupted by a variety of mechanisms leading to an accumulation of aberrant transcripts and a premature termination codon (Wang et al. 2015). Defects of alternative splicing gene expression have been reported in various human pathologies including hearing impairment (Alasti et al. 2008; Seiler et al. 2018).

Although splice variants are common in hearing impairment, the way in which variation alters exon recognition and thus protein synthesis can vary (Chai et al. 2014; Ahmed et al. 2021). For instance, a splicing variant of *OTOF* (NM_194248, c.3289-1G > T) was found to segregate with a profound hearing impairment phenotype in a Pakistani family. This single base pair substitution resulted in the deletion of 10 bp (splicing variant 1) or 13 bp (splicing variant 2) of exon 27, and subsequently resulted in truncated proteins of 1141 and 1140 amino acids, respectively (Ahmed et al. 2021).

Here, we revealed, using the powerful minigene assay, that the c.550-6A > G variant (variant NM_001042702.5) did not alter the canonical acceptor splice site; which remained conserved here. Instead, the variant created a new acceptor site (− 6 down the sequence) which appears to be more efficient than the common acceptor site. Indeed, sequencing data showed that this inclusion of five nucleotides after the splice site led to alternative splicing resulting in the appearance of a premature stop codon and thus a truncated protein. (p. Phe184Tyrfs*26) (Fig. 2B and C).

As a result, we can assume that the hearing impairment diagnosed in our patient was caused by the formation of a truncated PJVK protein through alteration of the transcript splicing site. Similarly, a splice-site donor variant in intron 2 (c.211 + 1G > T) of *PJVK* gene has been reported to be pathogenic and responsible for ARNSHI, via a truncated PJVK protein in the Iranian population (Sloan-Heggen et al. 2015). To our knowledge, apart from two variants leading to a truncated *PJVK* protein (c.113dep, p.Lys41Glufs*8 and c.406C > T, p.Arg92*) (Table 3) in a Moroccan family with a severe to profound deafness, no other *PJVK* variant has been reported in both the North African and sub-Saharan populations.

The occurrence of a premature stop codon, as in our case, may have induced either the formation of a truncated polypeptide or no protein synthesis at all due to nonsense-mediated mRNA decay (NMD). Both situations can lead to the hearing impairment phenotype observed in our patient, as a result of an incomplete and non-functional protein primary structure or no protein, respectively.

Variations which generate premature codon stop activating NMD have been linked to various diseases such as Duchenne muscular dystrophy (DMD) which affects myofibers where the absence of expression of the full-length dystrophin triggered major transcriptomic and functional abnormalities in myoblasts (García-Rodríguez et al. 2020).

## Conclusion

We have linked autosomal recessive hearing impairment in a Mauritanian family to the DFNB59 locus and identified a novel biallelic predicted pathogenic variant by targeted exome sequencing, Sanger sequencing, and minigene approaches.

In three previous studies, we detected the *GJB2* (MIM 121011) and *LRTOMT* (MIM 611451) variants and *CDC14A* (MIM608653) variants implicated in hearing impairment in the Mauritanian population.

This study revealed an additional report suggesting that splice-site variants can lead to DFNB59-associated ARNSHI in which the protein exerts a novel deleterious action.

Our variant is only the second report of pathogenic splice-site variants in *PJVK* gene leading to severe to profound non-syndromic hearing impairment.

## Data Availability

The datasets used and/or analyzed during the current study are available from the corresponding author on reasonable request.
